# Diversity of endophytic bacteria of mulberry (*Morus* L.) under cold conditions

**DOI:** 10.3389/fmicb.2022.923162

**Published:** 2022-07-19

**Authors:** Chuan-jie Chen, Guang Guo, Meng Li, Xiao-yan Liang, Yin-yu Gu

**Affiliations:** Shandong Institute of Sericulture, Shandong Academy of Agricultural Sciences, Yantai, China

**Keywords:** mulberry, endophytic, bacteria, diversity, cold condition

## Abstract

Endophytic bacteria are known to impact the growth and fitness of agriculturally relevant plants. However, there are limited reports describing endophytic bacteria related to mulberry (*Morus* L.). The present study used Illumina-based 16S rRNA gene sequencing to investigate the endophytic bacterial communities of two mulberry cultivars with differing resistance to low temperature, under cold conditions. In most cases, the bacterial communities of endophytes in the root exhibited higher richness compared with those in the stem, and the communities in resistant cultivar X792 exhibited higher richness compared with those of the sensitive cultivar “Da Shi” (DS). The difference in the proportion of unique operational taxonomic units showed the same trend. The number of genera with significant differences in abundance was greater between organs than between months, and greater between months than between cultivars. Microbial diversity analysis showed that Proteobacteria and Actinobacteria were the dominant phyla in all samples, while *Pseudomonas*, *Steroidobacter*, and *Rhodococcus* were the dominant genera in different samples. There were significant differences between cultivars DS and X792 in the relative abundance of *Pseudomonas*, *Acidibacter*, *Frigoribacterium*, *Gaiella*, and *Pseudokineococcus*. PICRUSt predictions indicated that the relative abundances of endophytic bacteria in membrane transport and signal transduction were significantly higher in the stem of resistant cultivar X792 in January compared with that of sensitive cultivar DS. Analysis of *β*-Diversity also revealed distinct differences in endophytic bacterial communities of stem and root, and communities of the stem in January and February. The complex correlation of the endophytic communities was higher in sensitive mulberry cultivar DS compared with resistant cultivar X792, in the stem compared with the root, and in January compared with February. Overall, findings from this study suggested that the diversity and community structure of endophytic bacteria in mulberry were significantly influenced by organs and months, followed by the host cultivar. The study provides insight into the complex microbial diversity of mulberry under cold conditions.

## Introduction

Low-temperature stress leads to a series of molecular, biochemical, physiological, and morphological changes that adversely affect plant growth and productivity, and it is a major factor limiting the productivity of agricultural crops. Cold-resistant microorganisms play an important role in agricultural production by promoting nitrogen fixation, nutrient accumulation, and growth, and inhibiting harmful pathogens and insects (Barka et al., 2006). Many cold-tolerant, potential plant-growth-promoting bacteria (PGPB) including *Arthrobacter*, *Bacillus*, *Exiguobacterium*, *Pseudomonas*, and *Providencia* etc. have been reported from low-temperature environments (Mishra et al., 2011; Selvakumar et al., 2011; Bisht et al., 2013; Yadav et al., 2014, 2015, 2016).

Endophytes are a class of microorganisms that live in various groups of healthy plants and have a harmonious relationship with the host plant in the intercellular spaces or cells of the organs (Xie and Xia, 2008). Although De Bary (1866) put forward the concept of endophytes as early as 160 years ago, research on endophytes has only attracted attention for recent decades. Studies demonstrated that plant endophytes can promote plant growth and development (Hallmann et al., 1997), and enhance host resistance to biotic and abiotic stress (whether drought, salt, or cold/thermal stress; Bacon et al., 2015). When plants are in contact with a microbe, regardless of fungi or bacteria, either a pathogen or a mutualist, is in part correlated with an increase in antioxidant or osmolyte concentrations and/or in the activities of antioxidant enzymes (Singh et al., 2011; Chen et al., 2014; Harman et al., 2021; Zhou et al., 2021). Furthermore, some endophytic bacteria were shown to be involved in tolerance to low-temperature stress (Barka et al., 2006; Fernandez et al., 2012; Theocharis et al., 2012), such as *Burkholderia phytofirmans PsJN* (Barka et al., 2006) and *Clavibacter* sp. strain Enf12 (Ding et al., 2011), which can promote the growth, stimulate physiological activity and improve plant tolerance to chilling stress through enhancing the antioxidant defense system. 16S rRNA functional prediction demonstrated that hosts that adapted to lower temperatures recruited endophytic communities with a higher abundance of genes related to cold resistance (Wei et al., 2021).

Mulberry, belonging to the genus *Morus* of the family Moraceae, is a species native to China and has been widely cultivated in many regions including Asia, Africa, America, and Europe (Khan et al., 2013). Mulberry is one of the major commercialized, perennially grown tree plants and is cultivated worldwide mainly for its foliage, which is the food of the silkworm (*Bombyx mori* L.) that produces the cocoon for silk (Shukla et al., 2016). In many mulberry-growing countries, particularly India and China, mulberry is used to feed the silkworm, but in most European countries, including Turkey and Greece, mulberries are grown for fruit production rather than foliage (Ercisli and Orhan, 2007). Freezing or extremely low temperatures are key factors influencing plant growth, development, and productivity in temperate regions. Frost and low temperature adversely affect mulberry leaf production, which ultimately affects silkworm rearing. Some research revealed a correlation between mulberry cold resistance and genes (Ukaji et al., 2004; Checker et al., 2011; Saeed et al., 2016). However, there is currently limited information available regarding the endophytic bacterial community of the mulberry under cold conditions. Thus, this study aimed to explore the relationship between microorganisms and low-temperature resistance by examining the endophytic microbial diversity of mulberry trees under cold conditions. Two mulberry cultivars with different resistance to low temperature were selected according to the frozen shoot rate (Frozen withered shoot length/Total shoot length; Pan and Zhang, 2006), and the endophytic bacteria of the stem and root of resistant cultivar X792 and sensitive cultivar DS in January and February were analyzed. To the best of our knowledge, this is the first study to characterize endophytes related to the mulberry under cold conditions, and findings from the study provide new insights into this bacterial community and lay a foundation for future studies.

## Materials and methods

### Sample collection and surface sterilization

Two mulberry cultivars, “Da Shi” (DS) and “Xuan792” (X792), were used in the study. X792 is resistant to low temperature (frozen shoot rate 5.06%), while DS is sensitive to low temperature (frozen shoot rate 30.43%). Both varieties were sampled at the experimental farm of Shandong Sericulture Research Institute (37°08′25.44′N, 121°08′33.98′E) in Yantai, Shandong Province, China, and shared the same climatic conditions. The local area has a temperate maritime climate, possessing a mean air temperature of 14°C and average annual rainfall of approximately 700 mm, with a frost-free period of more than 210 days, and an annual sunshine duration of more than 2,100 h. The maximum and the minimum temperatures in January were 5.55 and −1.97°C, respectively, and in February were 6.97 and −0.10°C, respectively. The temperature of the sampling day in January was −4~3°C, and in February was 3~9°C.

Three replicates for each sample (2 cultivars × 2 months× 2 organs) were performed in the study. The samples were collected from 10-year-old trees of mulberry cultivars, with three healthy-looking, medium-growth plants randomly selected from each site in January and February of 2019. Plants were at least 2 m apart within an area of 100 m^2^. Healthy branches approximately 180.0 cm in length and 1.5–1.8 cm in diameter, and roots approximately 30.0 cm in length and 0.3–0.8 cm in diameter, were collected from the three individual plants of each cultivar. Whole branches and roots were placed in Ziploc bags and stored at 4°C during transportation to the laboratory, then were processed within 24 h of collection. Branches and roots were washed in running tap water to remove surface debris and then the middle 60 cm of the branches were cut into several 5.0-cm segments, while the middle 18 cm of the roots were cut into several 2.0-cm segments (15–20 cm depth). Six surface-sterilized segments of each cultivar were randomly selected, pooled, and served as one replicate for further endophyte enrichment (Nxumalo et al., 2020).

Plant materials were surface sterilized using the procedure of Du et al. (2020). Briefly, the stem and root were washed thoroughly with sterile water, then immersed in 70% ethanol for 3 min, washed with fresh sodium hypochlorite solution (2.5% available Cl^−^) for 5 min with agitation, rinsed three times with 70% ethanol for 30 s, and finally washed five times with sterile distilled water. The sterile distilled water used in the final wash was cultivated to determine the success of the surface disinfection. Briefly, 100 μl of the final rinse water was plated on an LB medium and examined for bacterial growth after incubation at 30°C for 72 h. If there was no bacterial growth, the surface-sterilization procedure was confirmed to be effective and the samples were used for further analysis.

### DNA extraction and PCR amplification

Genomic DNA of the microbial community of the mulberry plants was extracted from stems and roots using the E.Z.N.A.® soil DNA Kit (Omega Bio-tek, Norcross, GA, United States) according to the manufacturer’s instructions. The quality of the extracted DNA was checked on a 1% agarose gel, and DNA concentration and purity were determined with a NanoDrop 2000 UV–vis spectrophotometer (Thermo Scientific, Wilmington, DE, United States). The hypervariable region V5–V7 of bacterial 16S rRNA genes was amplified by PCR using primer pairs 799F (5′-AACMGGATTAGATACCCKG-3′) and 1193R (5′-ACGTCATCCCCACCTTCC-3′; Beckers et al., 2017). The PCR mixtures contained 4 μl 5× *TransStart*FastPfu buffer, 2 μl of 2.5 mM dNTPs, 0.8 μl each primer (5 μM each), 0.4 μl *TransStart*FastPfu DNA Polymerase, 10 ng template DNA, and ddH_2_O to 20 μl, and reactions were performed in triplicate. PCR cycling conditions comprised an initial denaturation at 95°C for 3 min, 27 cycles of denaturing at 95°C for 30 s, annealing at 55°C for 30 s, and extension at 72°C for 45 s, followed by a single extension at 72°C for 10 min and a continued hold at 4°C. The complete sequences generated in this study are available in the NCBI SRA database under accession number SRR18790631–SRR18790654.

### Illumina MiSeq sequencing and processing of sequencing data

The resulting PCR products were extracted from 2% agarose gels, purified using the AxyPrep DNA Gel Extraction Kit (Axygen Biosciences, Union City, CA, United States) according to the manufacturer’s instructions, and quantified using a Quantus™ Fluorometer (Promega, United States). Purified amplicons were pooled in equimolar ratios and paired-end sequenced by Majorbio Bio-Pharm Technology Co. Ltd. (Shanghai, China) using an Illumina MiSeq PE300 platform (Illumina, San Diego, CA, United States) according to standard protocols.

Raw 16S rRNA gene sequencing reads were demultiplexed, quality-filtered by fastq version 0.20.0 (Chen et al., 2018), and merged by FLASH version 1.2.7 (Magoč and Salzberg, 2011). Operational taxonomic units (OTUs) with a 97% similarity cut-off (Stackebrandt and Goebel, 1994; Edgar, 2013) were clustered using UPARSE version 7.1 (Edgar, 2013), and chimeric sequences were identified and removed. The taxonomy of each OTU representative sequence was analyzed by RDP Classifier version 2.2 (Wang et al., 2007) against the 16S rRNA database using a confidence threshold of 0.7 (Beckers et al., 2016).

### Data analysis

Bacterial relative abundance, α-diversity, community composition, β-diversity, network structure, and functional analysis were performed using the free online Majorbio Cloud Platform.^1^ Alpha diversity was calculated including Chao, ACE, Shannon, and Simpson indices using Mothur software (versionv.1.30.2). Rarefaction curves were also generated using Mothur at a 97% identity level. The Venn, Bar, and Heatmap diagram was generated using R script (version 3.3.1), and Silva (Release138; http://www.arb-silva.de) was used for taxonomic classification. β-diversity was visualized using principal coordinates analysis (PCoA) and non-metric multidimensional scaling (NMDS) based on the distance matrix, with the calculation of the Euclidean and Bray-Curtis algorithm, respectively. The potential function of bacterial communities was predicted using PICRUSt2 (Douglas et al., 2020; Phylogenetic Investigation of Communities by Reconstruction of Unobserved States), and the histogram was created using Graphypad Prism 9.3.1. The OTU abundance table was first normalised by PICRUSt2 (the PICRUSt process stores the COG information and KO information corresponding to the OTU), i.e., the influence of the number of copies of the 16S marker gene in the species genome was removed; then obtain the COG family information and KEGG Ortholog (KO) information corresponding to the OTU, and the abundance of each COG and the KO abundance were calculated; According to the information in the COG database, the description information of each COG and its function information can be parsed from the eggNOG database, so as to obtain the functional abundance spectrum; based on the information in the KEGG database, the KO, Pathway, and EC information can be obtained, and the abundance of each functional class can be calculated based on the OTU abundance. In addition, for Pathway, three levels of metabolic pathway information can be obtained by using PICRUSt2, and the abundance table of each level can be obtained respectively, significant differences were analyzed by TTEST in excel. The community differences for the 10 most abundant bacterial genera distributions were evaluated using the Student’s *T* and one-way ANOVA, with values of *p* < 0.05 considered statistically significant. Finally, a network analysis was performed by spearman using Network × software to explore the complexity of the interactions among the microbial taxa, the absolute value of the correlation coefficient ≥0.7, *p* < 0.05, and the diagram was drawn by Cytoscape 3.5.1. All experiments were conducted in triplicate. Data are presented as means with SDs. Statistical analyses were performed using DPS Statistics 18.10 software.^2^ Differences between the means of different treatments were determined using the Duncan test at *p* < 0.05. The OTU table with taxonomic annotations was provided as Supplementary Table S1.

## Results

### Sequence data and α-diversity index analysis

After read-quality filtering, a total of 1,059,743 high-quality sequences remained and were queried. The total number of bases was 399,174,839, and the average read length was 376.63 bp, (Supplementary Table S2). Rarefaction curves (Supplementary Figure S1), combined with the estimated coverage values (Table 1), suggested that the data were sufficiently large to capture most of the bacterial diversity in the samples. The number of OTUs obtained was highest in the stem of X792 in February, followed by that in the root of X792 in January, while the lowest number of OTUs was present in the stem of X792 in January.

**Table 1:** Operational taxonomic unit (OTU; 97% similarity cut-off) richness and diversity indices of different samples associated with mulberry.

Sample	OTUs observed	Shannon	Simpson	ACE	Chao	Coverage (%)
JSDS	285 ± 11 cd	3.13 ± 0.32 a	0.14 ± 0.08 b	308 ± 30 cd	336 ± 52 e	99.82
JS792	250 ± 28 d	2.70 ± 0.59 ab	0.20 ± 0.11 b	270 ± 50 d	290 ± 80 e	99.85
JRDS	353 ± 78 c	2.83 ± 0.47 ab	0.16 ± 0.08 b	645 ± 382 b	562 ± 110 bc	99.38
JR792	451 ± 98 b	3.16 ± 0.40 a	0.12 ± 0.03 b	687 ± 198 b	662 ± 205 b	99.26
FSDS	286 ± 70 cd	1.38 ± 0.77 c	0.55 ± 0.24 a	345 ± 67 cd	346 ± 63 de	99.64
FS792	682 ± 148 a	1.75 ± 0.40 c	0.50 ± 0.09 a	1,309 ± 672 a	1,076 ± 277 a	98.56
FRDS	323 ± 101 cd	2.57 ± 0.70 b	0.21 ± 0.17 b	621 ± 324 b	501 ± 219 cd	99.42
FR792	350 ± 160 c	3.14 ± 0.76 a	0.11 ± 0.1 b	564 ± 139 bc	494 ± 216 cd	99.42

Data are means ± SD of three replicates. Values within a column followed by different lowercase letters are significantly different (*p* < 0.05). JSDS, stem of DS in January; JS792, stem of X792 in January; JRDS, root of DS in January; JR792, root of X792 in January; FSDS stem of DS in February; FS792, stem of X792 in February; FRDS, root of DS in February; and FR792, root of X792 in February.

**Figure 1:**
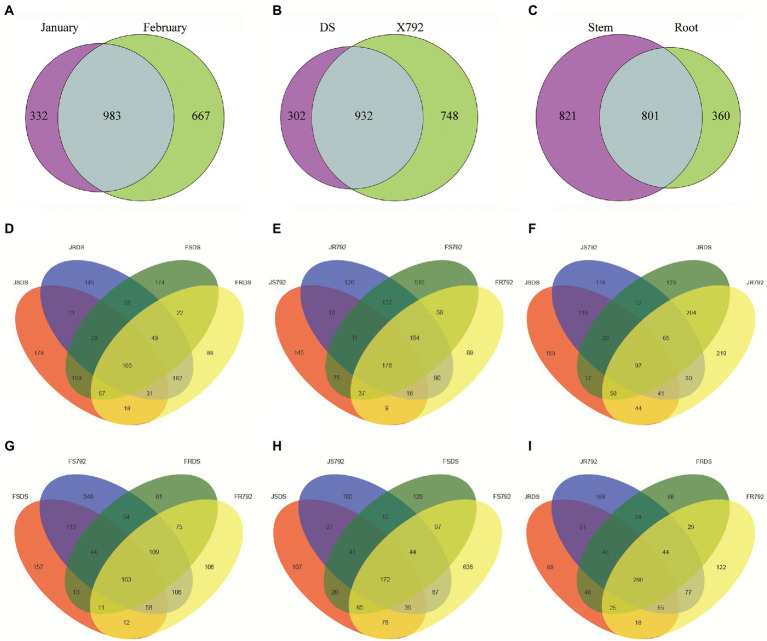
Venn diagrams of the number of operational taxonomic units (OTUs) obtained in different organs of different cultivars of mulberry in different months (January and Fabruary). Values represent the number of OTUs. **(A)** Grouping by month. January and February represent January bacterial communities and February bacterial communities of the two cultivars, respectively. **(B)** Grouping by cultivar. DS and X792 represent bacterial communities of cultivars “DS” and “X792” in 2 months, respectively. **(C)** Grouping by organ. Stem and root represent bacterial communities from stem and root of two cultivars in 2 months, respectively. **(D,E)** Grouping by different organs of DS **(D)** and X792 **(E)** in different months. **(F,G)** Grouping by different organs of different cultivars in January **(F)** and February **(G)**. **(H,I)** Grouping by stem **(H)** and root **(I)** of different cultivars in different months.

The number of common and unique bacterial OTUs in the different samples was presented in Venn diagrams (Figure 1). The numbers of shared OTUs between January and February samples (983, Figure 1A) and between DS and X792 samples (932, Figure 1B) were both higher than the number of shared OTUs between stem and root samples (801, Figure 1C). For unique OTUs, the number obtained in January (332) was lower than the number obtained in February (667; Figure 1A); the number in the sensitive mulberry cultivar DS (302) was lower than the number in the resistant mulberry cultivar X792 (748; Figure 1B); and the number obtained from the stems (821) was higher than the number obtained from the roots (360; Figure 1C).

In addition, the number of OTUs shared between different organs and months for the DS cultivar (103; Figure 1D) was lower than that of the X792 cultivar (176; Figure 1E); the number shared between different organs and cultivars in January (97; Figure 1F) was lower compared with that in February (163; Figure 1G); and the number of OTUs shared between different months and cultivars for the stem samples (172; Figure 1H) was lower than that obtained for the root samples (260; Figure 1I). The number of unique OTUs in the roots of the DS cultivar in February (FRDS, 88) was lower than those of the other samples of the DS cultivar (JSDS, 178; JRDS, 145; and FSDS,174; Figure 1D); but when compared within stems (Figure 1H), or within February (Figure 1G), or within X792 (Figure 1E), the number of unique OTUs in the stems of X792 in February was the highest (516 in Figure 1E; 548 in Figure 1G; 636 in Figure 1H); and when compared within roots (Figure 1I), or within January (Figure 1F), the number of unique OTUs in the roots of X792 in January was the highest (219 in Figure 1F; 189 in Figure 1I).

These data suggest that month, mulberry cultivar., and organ all contributed to the observed variation in the composition of the mulberry endophytic bacterial OTUs.

### Microbial taxonomic analysis at the phylum level

**Figure 2:**
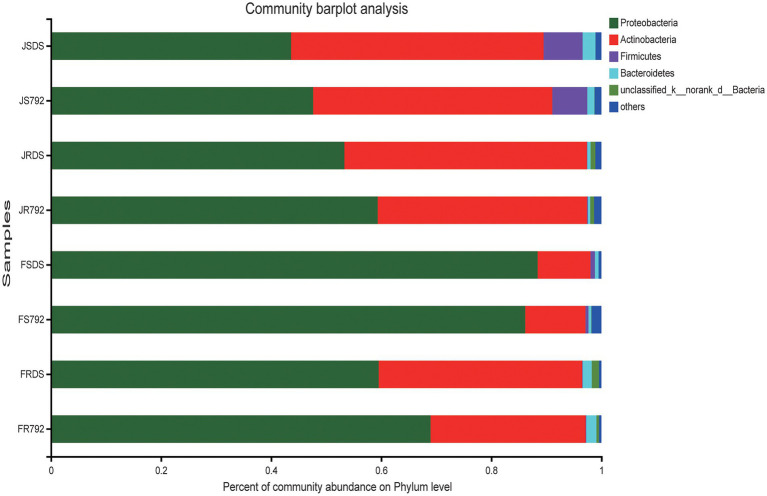
Relative abundance of endophytic bacteria from different communities at the phylum level. Taxa with an abundance <0.01 are included in “others.” The *x*-axis represents different communities (month × organ × cultivar), and the *y*-axis represents the relative abundance of all communities.

The obtained sequences were classified into 25 phyla, 56 classes, 169 orders, 325 families, and 647 genera. The bacterial composition and relative abundances varied across different samples. Figure 2 showed the diversity of bacterial communities in different samples at the phylum level. The dominant bacterial phyla were Proteobacteria and Actinobacteria across all samples; Actinobacteria was the predominant phylum in JSDS (45.88%), while Proteobacteria (47.65–88.44%) was the predominant phylum across all other samples. The relative abundance of Proteobacteria was higher in all cultivar X792 samples compared with the DS samples, except for the stem in February, while Actinobacteria showed the opposite trend. For Proteobacteria, Actinobacteria, and Firmicutes, there was no significant difference in relative abundance between mulberry cultivars; while there was a significant increase over relative abundance of Actinobacteria and Firmicutes in the stem of January compared with February (*p* < 0.01), and Proteobacteria showed the opposite trend, but not in root samples. Proteobacteria showed a significant increase in stems compared with roots in February (*p* < 0.01), and Actinobacteria showed the opposite trend, and Firmicutes showed significant increase in stems compared with roots in January (*p* < 0.01; Supplementary Table S3). These analyses indicated that the temperature had a greater influence on the endophytic bacterial content of the stem compared with that of the root.

### Microbial taxonomic analysis at the genus level and core genus distribution

**Figure 3:**
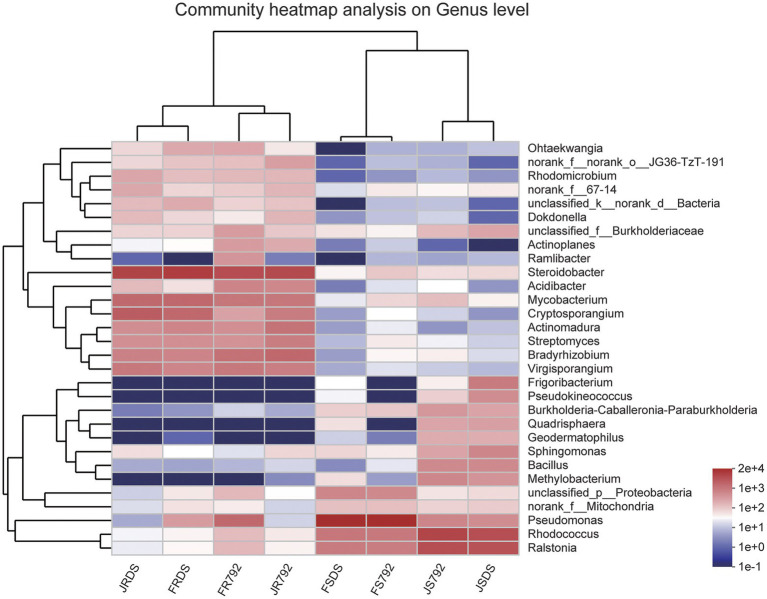
Heatmap displaying the relative abundances of the predominant genera (top 30) in each sample. The dendrogram represents complete-linkage agglomerative clustering, based on Euclidean dissimilarities.

Clustering of the top 30 genera was shown in Figure 3 (with additional supporting data present in Supplementary Table S4). These 30 bacterial genera belonged to five phyla including Proteobacteria (15 genera), Actinobacteria (12), Firmicutes (1), Bacteroidetes (1), and unclassified_k_norank_d_Bacteria (1). *Pseudomonas*, *Steroidobacter*, *Rhodococcus*, *Ralstonia*, *Mycobacterium*, and *Cryptosporangium* were the most abundant (>0.1%) genera across all samples, but the genera distributions differed greatly across different samples. *Pseudomonas* was the predominant genus in FSDS and FS792, while *Steroidobacter* was the most abundant genus in JRDS, JR792, FRDS, and FR792, and *Rhodococcus* was the predominant genus in JSDS and JS792. These results showed that the most predominant genus of the stem in January was *Rhodococcus*, and in February was *Pseudomonas*, while *Steroidobacter* was the predominant genus in the root, and there was no significant difference in the relative abundance of genus between mulberry cultivars. The clustering analysis showed a clear similarity among samples from each tissue (roots are similar and shoots are similar). For roots, two cultivars were gathered separately, and for stems, there were more similarities in January, and the same rule occurred in February.

**Figure 4:**
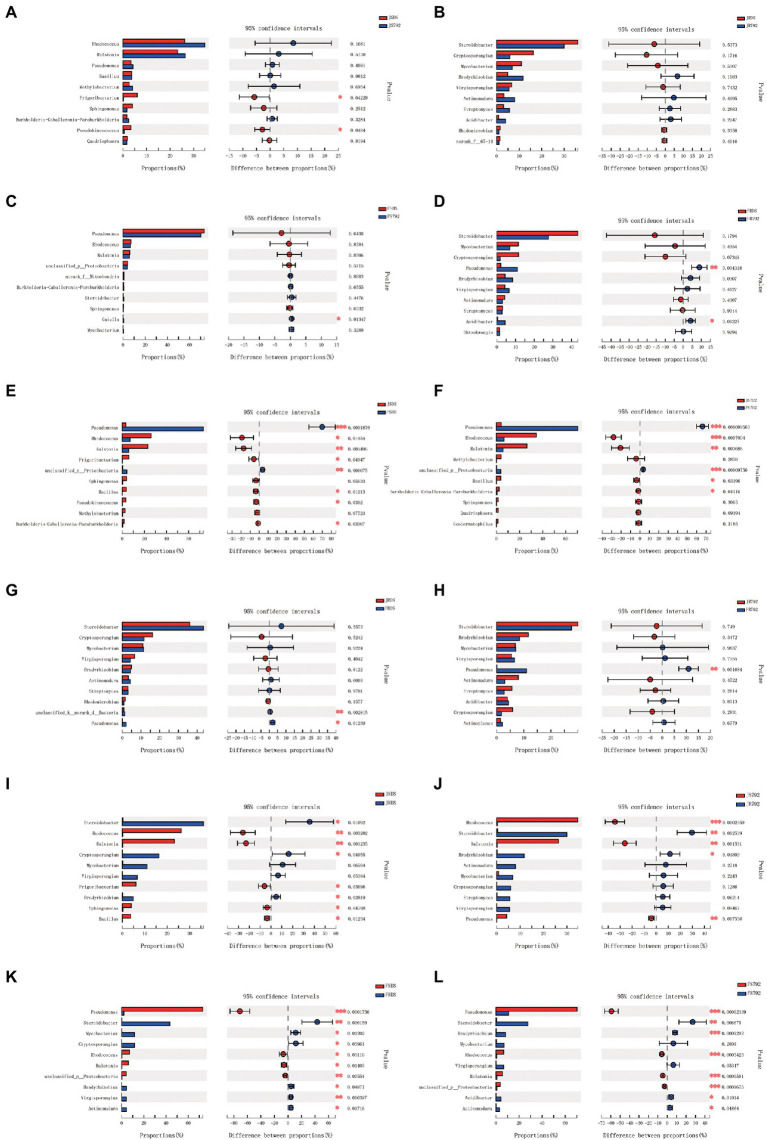
Comparison of the abundance of the top 10 dominant bacterial genera in different samples. *** indicates a significant difference at *p* < 0.001, ** *p* < 0.01, and * *p* < 0.05. The *x*-axis represents the mean proportion of the genus and the *y*-axis shows the top 10 dominant bacterial genera. **(A)** Compared between different cultivars of stem in January. **(B)** Compared between different cultivars of root in January. **(C)** Compared between different cultivars of stem in February. **(D)** Compared between different cultivars of root in February. **(E)** Compared between different months of DS stem. **(F)** Compared between different months of X792 stem. **(G)** Compared between different months of DS root. **(H)** Compared between different months of X792 root. **(I)** Compared between different organs of DS in January. **(J)** Compared between different organs of X792 in January. **(K)** Compared between different organs of DS in February. **(L)** Compared between different organs of X792 in February.

The relative abundance of the top 15 core genera was also compared, and some differences were observed (Figure 4). The relative frequencies of *Frigoribacterium* (*p* = 0.04) and *Pseudokineococcus* (*p* = 0.04) were higher in JSDS compared with those in JS792 (Figure 4A), but there was no significant difference between JRDS and JR792 (Figure 4B), while that of the genus *Gaiella* (*p* = 0.013) was significantly lower in FSDS compared with that in FS792 (Figure 4C). The relative abundances of *Pseudomonas* (*p* = 0.004) and *Acidibacter* (*p* = 0.012) were significantly lower and significantly lower, respectively, in FRDS compared with those in FR792 (Figure 4D).

The relative abundance of *Pseudomonas* was also significantly lower in JSDS compared with that in FSDS (*p* = 0.0002), while that of *Ralstonia* was significantly higher in JSDS compared with that in FSDS (*p* = 0.005; Figure 4E). Moreover, the relative abundances of *Pseudomonas* (*p* = 0.000009) and *Rhodococcus* (*p* = 0.0007) were significantly lower and significantly higher, respectively, in JS792 compared with those in FS792, and that of *Ralstonia* was also significantly higher in JS792 compared with that in FS792 (*p* = 0.004; Figure 4F). *Pseudomonas* had a significantly lower abundance in JRDS compared with that in FRDS (*p* = 0.012; Figure 4G), and had a significantly lower relative abundance in JR792 compared with that in FR792 (*p* = 0.002; Figure 4H). The relative abundances of *Rhodococcus* (*p* = 0.003) and *Ralstonia* (*p* = 0.001) were significantly higher, and that of *Steroidobacter* was significantly lower, in JSDS compared with JRDS (*p* = 0.01; Figure 4I). In addition, the relative abundance of *Rhodococcus* was significantly higher in JS792 compared with that in JR792 (*p* = 0.0003), while that of *Steroidobacter* was significantly lower (*p* = 0.003), and those of *Ralstonia* (*p* = 0.002) and *Pseudomonas* (*p* = 0.008) were significantly higher, in JS792 compared with JR792 (Figure 4J). Furthermore, the genus *Pseudomonas* had a significantly higher relative abundance in FSDS compared with that in FRDS (*p* = 0.0002), while relative abundances of *Steroidobacter* (*p* = 0.006) and *Virgisporangium* were significantly lower in FSDS compared with those in FRDS (*p* = 0.006; Figure 4K). Finally, the relative abundances of *Pseudomonas* (*p* = 0.00002), *Rhodococcus* (*p* = 0.0005), and *Ralstonia* (*p* = 0.00005) were significantly higher, and that of *Bradyrhizobium* (*p* = 0.0009) was significantly lower, in FS792 compared with FR792, and that of *Steroidobacter* (*p* = 0.007) was significantly lower in FS792 compared with that in FR792 (Figure 4L). Overall, there were more significant differences in the relative abundances of the top 15 core genera between stem and root, especially in February, followed by the stem in January and February, while there were fewer significant differences between cultivars.

### β-Diversity analysis

**Figure 5:**
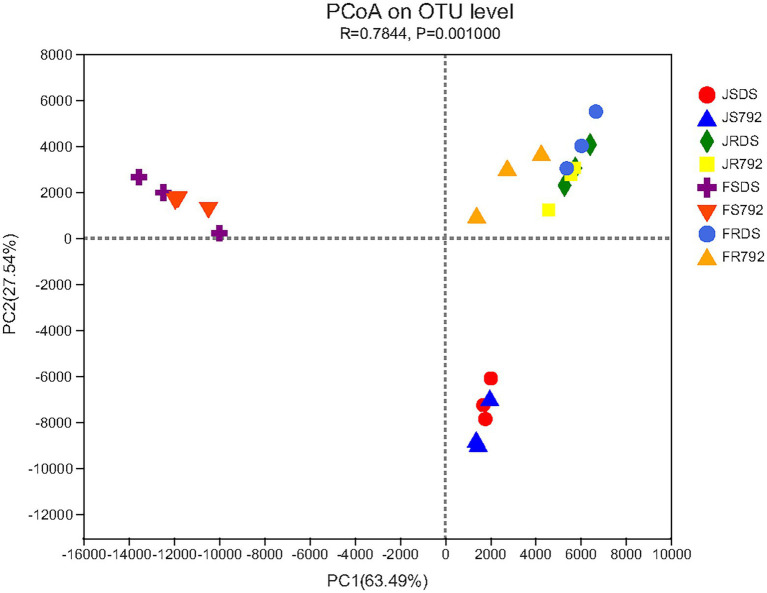
Principal coordinates analysis (PCoA) plot of the relationship between samples based on similarity in the community composition of bacterial OTUs. Two first components (PC1 and PC2) were plotted and represent 91.03% of the variation.

To further compare the relationship of endophytic bacteria populations among the stem and root of two mulberry cultivars in January and February, principal coordinates analysis (PCoA) based on Euclidean distances with arithmetic mean clustering was conducted using the genera. This analysis revealed the main variations in bacterial community composition and abundance among the samples. The PCoA results graphically demonstrated that organs or months were strong factors in accounting for the observed variations in the composition of the endophytic bacterial community, in which samples of the stem in January were placed at a higher PC 1 value (63.49%), while samples of the stem in February appeared a higher PC 2 value (27.54%), and root samples showed higher values of both PC 1 and PC 2 (Figure 5), and *p* = 0.001. Samples of the roots of different cultivars in different months clustered together, but the stems showed some separation, with samples of the stems in January and February clustering together, respectively. The PCoA results were supported by non-metric multidimensional scaling (NMDS) plots (*p* = 0.001; Supplementary Figure S2). In summary, these analyses revealed distinct differences in endophytic bacterial communities of stem and root, and stem of January and February, while no clustering was evident due to cultivars or root of January and February. This finding confirmed the results for the differential analysis of genera.

### Network structure

**Figure 6:**
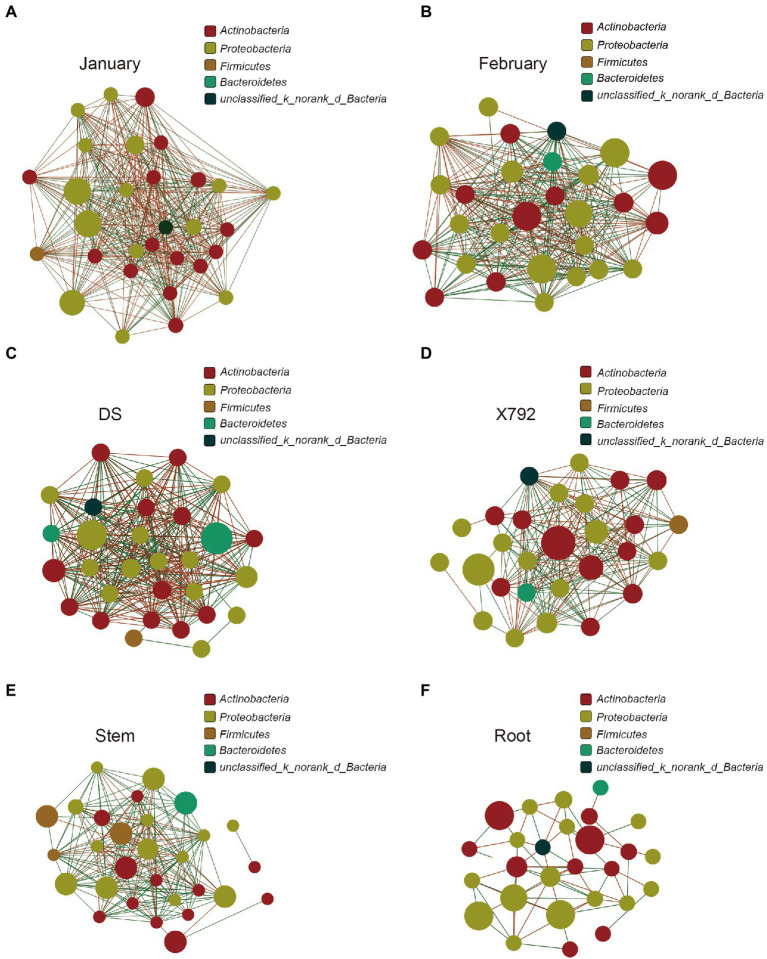
Correlation network analysis of microbial communities in January **(A)**, February **(B)**, DS **(C)**, X792 **(D)**, stem **(E)**, and root **(F)**. Node color represents phylum classification. The size of the node is proportional to the richness of bacteria. Edge color corresponds to positive (red) and negative (green) correlations, and the edge thickness is equivalent to the correlation values.

To explore the complexity of the interactions within the endophytic communities among the different samples, a correlation network analysis was conducted, and its topological properties were calculated. The correlation network analysis revealed that there was a difference between January and February. The complexity and modular structure were higher in January (Figure 6A) compared with that in February (Figure 6B), specifically, the average number of connections per node was higher in January (node average degree = 26.60) relative to the February samples (node average degree = 21.57; Table 2). Furthermore, January also presented a higher number of negative correlations (negative edges = 210) compared with February (negative edges = 113) on the basis of the same number of positive correlations. Additionally, the correlation network analysis of microbial communities for the sensitive or resistant cultivar also revealed differences. The sensitive mulberry cultivar DS (Figure 6C) appeared to have a more complex correlation compared with the resistant mulberry cultivar X792 (Figure 6D). The node average degree, positive edges, and negative edges in DS (node average degree = 22.07, positive edges = 163, and negative edges = 168) were all higher than those in X792 (node average degree = 15, positive edges = 120, and negative edges = 90).

**Table 2:** Correlation network analysis of microbial communities.

	January	February	DS	X792	Stem	Root
Node numbers	30	28	30	28	29	28
Edge numbers	399	302	331	210	218	62
Node average degree	26.6	21.57	22.07	15	15.03	4.43
Positive edges	189	189	163	120	160	30
Negative edges	210	113	168	90	58	32

The correlation network indices were calculated based on the top 30 genera. The average number of connections per node in the network, that is, the node connectivity.

The correlation network analysis was performed to assess the complexity of the interactions among the microbial taxa, the results also revealed a strong difference between the communities based on organs, such as stem (Figure 6E) and root (Figure 6F). The edge number for stem (edge = 218) was much higher compared with that of root (edge = 28), with 130 positive edges (positive edges of stem = 160, positive edges of root = 30) and 26 negative edges (negative edges of stem = 58, negative edges of root = 32). Simultaneously, the average number of connections per node was also much higher in the stem (node average degree = 15.03) compared with that in the root (node average degree = 4.43). In summary, the correlation network indicated that the interaction degree of the microbial community of mulberry was strongly influenced by the month, cultivar resistance, and organs. January, sensitive mulberry cultivar., and stem possessed a greater microbial complexity and abundance compared with February, the resistant mulberry cultivar., and root. The nodes with the highest connections in January were *Quadrisphaera*, *Steroidobacter*, *Pseudokineococcus*, *Dokdonella*, *Streptomyces*, *Rhodomicrobium*, *Methylobacterium*, *Virgisporangium*, *Frigoribacterium*, and *unclassified_k__norank_d__Bacteria* with a degree of 29, and in February was *unclassified_p__Proteobacteria, unclassified_k__norank_d__Bacteria*, and *Pseudomonas* with the degree of 26. *Burkholderia-Caballeronia-Paraburkholderia, and unclassified_k__norank_d__Bacteria* were the genera with the highest degree of 23 in cultivar X792, and *Methylobacterium* was the genus with the highest degree of 23 in cultivar DS. *Ralstonia* (degree = 10) and *Pseudomonas* (degree = 24) were the genera with the highest connections in root and stem, respectively.

### Potential functional consequences

**Figure 7:**
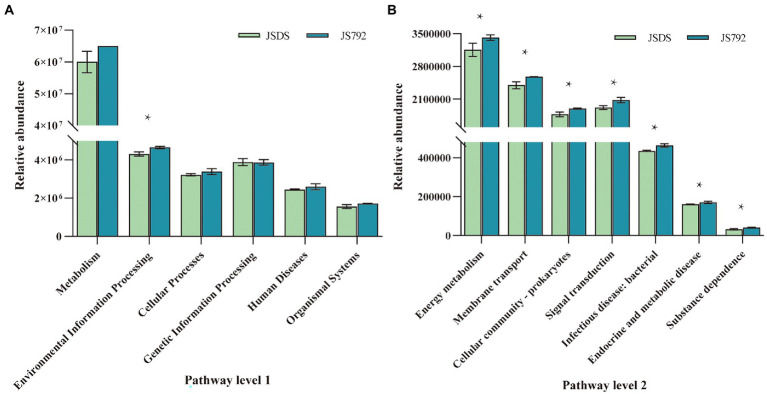
Functional analysis of microbial communities between mulberry cultivars. **(A)** Pathway level 1. **(B)** Pathway level 2. * indicates a significant difference at *p* < 0.05.

Functions of microbial communities from all samples were predicted using PICRUSt2 on level 1 and level 2 (Figure 7; Supplementary Tables S10, S11). Genes associated with environmental information processing (level 1), membrane transport, and signal transduction (level 2) were significantly (95% CIs, *p* < 0.05) more abundant in the stem of resistant cultivar X792 compared with the sensitive cultivar DS in January, which may be related to low-temperature resistance, and warrants further research. Furthermore, Welch’s *t*-test results (Supplementary Table S6) indicated that, except for the environmental information processing of stems in January, there were no significant differences in several predicted pathways (e.g., metabolism, environmental information processing, cellular processes, genetic information processing, human diseases, and organismal systems) among the other samples based on cultivars. In addition, there were no significant differences between the root of X792 in January and February for the five pathways on level 1, as well as the JRDS and FRDS for genetic information processing and organismal systems pathways, but there were significant or significant differences in the other samples based on months. Except for the genetic information processing pathway of the JS792 and JR792 samples, there were no significant differences among the other samples in January based on organs, but there were significant or significant differences throughout the five pathways in all samples in February. The stems of January and February had different degrees of significant differences in the five pathways, as did the stem and root of February. Overall, Environmental information processing was the pathway exhibiting the most differences, followed by the cellular process.

## Discussion

Microbial endophytes play an important role in the ecology, health, and growth promotion of plants (Lodewyckx et al., 2002; Mahajan and Tuteja, 2005; Hardoim et al., 2015; Vandenkoornhuyse et al., 2015; Hassani et al., 2018). However, despite the importance of endophytes, there are limited reports concerning endophytic microbial populations in mulberry. Therefore, the diversity of endophytic bacteria of mulberry under cold conditions was investigated in the present study using Illumina Miseq sequencing of the V5–V7 variable region of the bacterial 16S rRNA gene.

Except for the stem of resistant mulberry cultivar X792 in February, all of the bacterial richness in the stem of the other samples was lower compared with that in the root, but cultivar DS did not reach a significant level. This was not entirely consistent with previous findings that bacterial communities of endophytes in the root exhibit higher richness than those in the stem (Ma et al., 2013; Beckers et al., 2017; Tian and Zhang, 2017; Table 1). The difference of X792 in February may be caused by the cultivar and environmental variability; the temperature rose in February and approached the critical point of germination, which resulted in a substantial increase in microbial richness. For cultivars, X792 samples had greater bacterial richness compared with DS samples of root in January and stem in February. This suggests that the richness in the resistant mulberry cultivar was higher than that in the sensitive cultivar in most cases. Richness in the stem of X792 in February was much higher than that in January, while there was no significant change in richness in the stem of DS, which suggested that X792 reacted faster to high temperatures. Bacterial richness in the root of both cultivars in January was higher compared with that in February, albeit in different proportions, and this may be also caused by the temperature.

Previous studies showed that although most endophytic bacteria colonizing the host plant originate from the rhizosphere soil (Compant et al., 2010; Yang et al., 2017), some may originate elsewhere such as through colonization of the phyllosphere *via* aerosols (Fahlgren et al., 2010). It is hypothesized that endophytic bacteria colonize plants primarily through the root network *via* natural and artificial wound sites, root hairs, and epidermal junctions (Shi et al., 2014). In the current study, the root and stem shared about one-fifth to one-third of the OTUs, which may be migrating from root to stem (Supplementary Tables S7–S9). In January, the proportion of unique OTUs in the root of both cultivars was greater than that in the stem, while the opposite trend was observed in February. This may be due to the increased activity of microorganisms in the atmospheres as the temperature increases. In addition, the difference in the proportion of unique OTUs was much greater in resistant cultivar X792 compared with that in sensitive cultivar DS, and further research is required to determine whether this might be related to low-temperature resistance.

Proteobacteria have previously been reported as the predominant phylum of endophytic bacteria followed by Actinobacteria, and the richness of Proteobacteria in roots is higher than that in stems (Akinsanya et al., 2015; Tian and Zhang, 2017; Ou et al., 2019). Similar results were found in most samples of the current study, except in one January sample which the dominant bacterial phylum in the stem of sensitive cultivar DS was Actinobacteria, followed by Proteobacteria (Figure 2). This may be an individual case. The relative abundance of Proteobacteria was markedly higher than that of Actinobacteria in the stem of both cultivars in February, while there were no big differences among other samples; this difference might be caused by environmental variability.

The species composition of microbial communities can be affected by many factors. Hardoim et al. (2011) reinforced the importance of understanding the genetic and (bio)chemical mechanisms involved in the interplay between soil type, plant genotype, rhizosphere microbiome, plant growth, and plant health. The current study revealed that for the top 15 core genera, there were only five genera that had significant differences between the two mulberry cultivars. These genera were *Frigoribacterium* and *Pseudokineococcus*, which had significantly greater relative frequencies in the stem of sensitive cultivar DS compared with that in resistant cultivar X792, and *Gaiella*, which showed the opposite trend in the stem, *Pseudomonas* and *Acidibacter*, which also showed the opposite trend in the root (Figure 4). Egamberdieva (2009) revealed that *Pseudomonas* provided important benefits to plants by synthesizing phytohormones and improving host stress tolerance. *Pseudomonas* is a recognized psychrophile (Moyer and Morita, 2001), and well-characterized and reported from low-temperature environments (Yadav et al., 2016). *Frigoribacterium* also was one of the bacteria isolated from cold environments on Earth, such as permafrost, cold soils and deserts, glaciers, lakes, sea ice in the Arctic, Antarctic, and high mountains, as well as the deep sea, ice caves, and the atmospheric stratosphere etc. (Xin et al., 2013). *Acidibacter* was first isolated and identified by Falagán and Johnson (2014), from a pit lake in an abandoned metal mine in southwestern Spain. However, little has been reported on the function of *Pseudokineococc* and *Gaiella*. Additional work is required to elucidate whether the presence of the above five genera contributes to the low-temperature resistance. Ou et al. (2019) revealed that *Pantoea*, *Methylobacterium*, and *Pseudomonas* were the predominant genera in mulberry endophytic communities. *Pantoea* and *Methylobacterium* were not detected in the present study, and this may be caused by the sampling area and season. The number of genera with significant differences in abundance was greater between organs than between months, and greater between months than between cultivars. Among these genera, *Steroidobacter*, one of the dominant genera in the root (Tian and Zhang, 2017), and *Bradyrhizobium*, a nitrogen-fixing bacteria that has attracted a lot of attention (Saranraj et al., 2021), were more abundant in root than in stem, while *Rhodococcus* and *Ralstonia* were more abundant in stem compared with root. *Steroidobacter* also was the predominant genus in root both in January and February, but for the stem, *Rhodococcus* was the predominant genus in January and *Pseudomonas* was the predominant genus in February (Supplementary Tables S4, S5). Since abundant *Pseudomonas* and *Sphingomonas* were found to be harbored by aerosol (Fahlgren et al., 2010), enrichment of *Pseudomonas* in the stem endosphere may occur *via* dual origins, colonization of the rhizosphere, and/or stem stomatal colonization. *Rhodococcus* are gram-positive bacteria isolated from a variety of environments, such as soil and deep sea, and have a wide variety of species. *Rhodococcus* species are frequently studied because they possess multiple functions, and are meaningful for their environmental and industrial biotechnology applications (Abdelmohsen et al., 2010, 2014; Graça et al., 2015; Elsayed et al., 2017; Krivoruchko et al., 2019).

Correlation network analysis demonstrated that there was lower complexity in the network of the resistant cultivar X792 compared with sensitive cultivar DS, which was similar to the findings of Ou et al. (2019) but contrary to the observation of Mendes et al. (2018). Furthermore, the complexity in the network of the root was lower compared with stem, and lower in February compared with January.

Low temperature is a major environmental factor that limits plant growth, productivity, and distribution. To ensure optimal growth and survival, plants must respond and adapt to low-temperature stress using a variety of biochemical and physiological processes. The major detrimental effect of freezing is that it induces severe membrane damage, and this damage is largely due to the acute dehydration associated with freezing (Steponkus, 1984; Steponkus et al., 1993). Furthermore, manipulation of signal transduction was shown to be an important way to make plants survive at low temperatures (Kim et al., 2003; Solanke and Sharma, 2008). Congruent with these observations, the function predictions obtained with PICRUSt in the current study indicated that the relative abundance of membrane transport and signal transduction (level 2), which belong to environmental information processing (level 1), was significantly higher in the stem of resistant cultivar X792 in January compared with that of sensitive cultivar DS. Since PICRUSt predictions are based on the use of a limited database to search for functions, these predictions can be biased and further study is required to reach more accurate conclusions.

## Conclusion

This study is the first to elucidate the bacterial diversity and composition of mulberry under cold conditions using high-throughput sequencing methods. The endophytic bacterial community of the stem and root of two mulberry cultivars with differing resistance to low temperature was explored under cold conditions (January and February). Organ and month were found to play keys role in determining the diversity and community composition of endophytic bacteria in mulberry, followed by the host cultivar. *Pseudomonas*, *Steroidobacter*, and *Rhodococcus* were the predominant genera among the different samples, *Pseudomonas*, *Acidibacter*, *Frigoribacterium*, *Gaiella*, and *Pseudokineococcus* were genera that had significant differences in the relative abundance between cultivars DS and X792. There were different degrees of significant differences in five functional pathways between the stems of January and February, and between the stem and root of February samples. The relative abundance of endophytic bacteria that function as membrane transport and signal transduction was significantly higher in the stem of resistant cultivar X792 in January compared with that of sensitive cultivar DS. The present study significantly enhances understanding of the factors influencing the community structures of endophytic bacteria and lays the foundation for conducting research on the resistance of mulberry endophytes to low temperatures. Further studies are necessary to elucidate the low-temperature resistance properties or functional traits of mulberry endophytes and the functional roles of bacterial species in plant–microbe interactions in mulberry.

## Data availability statement

The datasets presented in this study can be found in online repositories. The names of the repository/repositories and accession number(s) can be found in the article/**Supplementary Material**.

## Author contributions

Y-yG contributed to the conception of the study and wrote the manuscript. C-jC, GG, ML, and X-yL performed the experiments and data analyses. All authors contributed to the article and approved the submitted version.

## Funding

This work was supported by the Sericultural Industry Technical System of Shandong Province (Grant no. SDAIT-18-09).

## Conflict of interest

The authors declare that the research was conducted in the absence of any commercial or financial relationships that could be construed as a potential conflict of interest.

## Publisher’s note

All claims expressed in this article are solely those of the authors and do not necessarily represent those of their affiliated organizations, or those of the publisher, the editors and the reviewers. Any product that may be evaluated in this article, or claim that may be made by its manufacturer, is not guaranteed or endorsed by the publisher.

## Footnotes

^1^ www.majorbio.com

^2^ www.dpsw.cn

## References

[ref1] Abdelmohsen U. R. Pimentel-Elardo S. M. Hanora A. Radwan M. Abou-El-Ela S. H. Ahmed S.. (2010). Isolation, phylogenetic analysis and anti-infective activity screening of marine sponge-associated Actinomycetes. Mar. Drugs 8, 399–412. doi: 10.3390/md8030399, PMID: 20411105 PMC2857355

[ref2] Abdelmohsen U. R. Yang C. Horn H. Hajjar D. Ravasi T. Hentschel U. (2014). Actinomycetes from red sea sponges: sources for chemical and phylogenetic diversity. Mar. Drugs 12, 2771–2789. doi: 10.3390/md12052771, PMID: 24824024 PMC4052315

[ref3] Akinsanya M. A. Goh J. K. Lim S. P. Ting A. S. Y. (2015). Metagenomics study of endophytic bacteria in *Aloe vera* using next-generation technology. Genom. Data 6, 159–163. doi: 10.1016/j.gdata.2015.09.004, PMID: 26697361 PMC4664749

[ref4] Bacon C. W. Palencia E. R. Hinton D. M. (2015). “Abiotic and biotic plant stress-tolerant and beneficial secondary metabolites produced by endophytic *Bacillus* species,” in Plant Microbes Symbiosis: Applied Facets (Chapter 8). (New Delhi: Springer), 163–177.

[ref5] Barka E. A. Nowak J. Clement C. (2006). Enhancement of chilling resistance of inoculated grapevine plantlets with a plant growth-promoting rhizobacterium, *Burkholderia phytofirmans* strain PsJN. Appl. Environ. Microbiol. 72, 7246–7252. doi: 10.1128/AEM.01047-06, PMID: 16980419 PMC1636148

[ref6] Beckers B. Beeck M. O. D. Thijs S. Truyens S. Weyens N. Boerjan W.. (2016). Performance of 16s rDNA primer pairs in the study of rhizophere and endosphere bacterial microbiomes in metabarcoding studies. Front. Microbiol. 7:650. doi: 10.3389/fmicb.2016.00650, PMID: 27242686 PMC4865482

[ref7] Beckers B. Beeck M. O. D. Weyens N. Boerjan W. V. Angronsveld J. (2017). Structural variability and niche differentiation in the rhizosphere and endosphere bacterial microbiome of field-grown poplar trees. Microbiome 5, 25. doi: 10.1186/s40168-017-0241-2, PMID: 28231859 PMC5324219

[ref8] Bisht S. C. Mishra P. K. Joshi G. K. (2013). Genetic and functional diversity among root-associated psychrotrophic Pseudomonad’s isolated from the Himalayan plants. Arch. Microbiol. 195, 605–615. doi: 10.1007/s00203-013-0908-4, PMID: 23861148

[ref9] Checker V. G. Chhibbar A. K. Khurana P. (2011). Stress-inducible expression of barley Hva1 gene in transgenic mulberry displays enhanced tolerance against drought, salinity and low temperature stress. Transgenic Res. 21, 939–957. doi: 10.1007/s11248-011-9577-8, PMID: 22160463

[ref10] Chen X. Song F. Liu F. Tian C. Liu S. Xu H. (2014). Effect of different arbuscular mycorrhizal fungi on growth and physiology of maize at ambient and low temperature regimes. Sci. World J. 2014:956141. doi: 10.1155/2014/956141 PMC4032736 24895680

[ref11] Chen S. F. Zhou Y. Q. Chen Y. R. Gu J. (2018). Fastp: an ultra-fast all-in-one FASTQ preprocessor. Bioinformatics 34, i884–i890. doi: 10.1093/bioinformatics/bty560, PMID: 30423086 PMC6129281

[ref12] Compant S. Clément C. Sessitsch A. (2010). Plant growth-promoting bacteria in the rhizo-and endosphere of plants: their role, colonization, mechanisms involved and prospects for utilization. Soil Biol. Biochem. 42, 669–678. doi: 10.1016/j.soilbio.2009.11.024

[ref13] De Bary A. (1866). Morphologie und Physiologie der Pilze, Flechten und Myxomyceten. Leipzig: W. Engelmann, 1–360

[ref14] Ding S. Huang C. L. Sheng H. M. Song C. L. Li Y. B. An L. Z. (2011). Effect of inoculation with the endophyte *Clavibacter* sp. strain Enf12 on chilling tolerance in *Chorispora bungeana*. Physiol. Plant. 141, 141–151. doi: 10.1111/j.1399-3054.2010.01428.x 21044086

[ref15] Douglas G. M. Maffei V. J. Zaneveld J. R. Yurgel S. N. Brown J. R. Taylor C. M.. (2020). PICRUSt2 for prediction of metagenome functions. Nat. Biotechnol. 38, 685–688. doi: 10.1038/s41587-020-0548-6, PMID: 32483366 PMC7365738

[ref16] Du J. J. Wang T. Zhou Q. X. Hu X. G. Wu J. H. Li G. F.. (2020). Graphene oxide enters the rice roots and disturbs the endophytic bacterial communities. Ecotoxicol. Environ. Saf. 192:110304. doi: 10.1016/j.ecoenv.2020.110304, PMID: 32066006

[ref17] Edgar R. C. (2013). UPARSE: highly accurate OTU sequences from microbial amplicon reads. Nat. Methods 10, 996–998. doi: 10.1038/nmeth.2604, PMID: 23955772

[ref18] Egamberdieva D. (2009). Alleviation of salt stress by plant growth regulators and IAA producing bacteria in wheat. Acta Physiol. Plant. 31, 861–864. doi: 10.1007/s11738-009-0297-0

[ref19] Elsayed Y. Refaat J. Abdelmohsen U. R. Fouad M. A. (2017). The genus *Rhodococcus* as a source of novel bioactive substances: a review. J. Pharm. Phytochem. 6, 83–92.

[ref20] Ercisli S. Orhan E. (2007). Chemical composition of white (*Morus alba*), red (*Morus rubra*) and black (*Morus nigra*) mulberry fruits. Food Chem. 103, 1380–1384. doi: 10.1016/j.foodchem.2006.10.054

[ref21] Fahlgren C. Hagström A. Nilsson D. Zweifel U. L. (2010). Annual variations in the diversity, viability, and origin of airborne bacteria. Appl. Environ. Microbiol. 76, 3015–3025. doi: 10.1128/AEM.02092-09, PMID: 20228096 PMC2863461

[ref590] Falagán C. Johnson D. B. (2014). *Acidibacter ferrireducens* gen. nov., sp. nov.: an acidophilic ferric iron-reducing gammaproteobacterium. Extremophiles 18, 1067–1073. doi: 10.1007/s00792-014-0684-3 25116055

[ref22] Fernandez O. Theocharis A. Bordiec S. Feil R. Jacquens L. Clement C.. (2012). *Burkholderiaphytofirmans* PsJN acclimates grapevine to low temperature by modulating carbohydrate metabolism. Mol. Plant Microbe Interact. J. 25, 496–504. doi: 10.1094/MPMI-09-11-0245, PMID: 22409157

[ref23] Graça A. P. Viana F. Bondoso J. Correia M. I. Gomes L. Humanes M.. (2015). The antimicrobial activity of heterotrophic bacteria isolated from the marine sponge *Erylus deficiens* (Astrophorida, Geodiidae). Front. Microbiol. 6:389. doi: 10.3389/fmicb.2015.00389, PMID: 25999928 PMC4423441

[ref24] Hallmann J. Quadt-Hallmann A. Mahaffee W. F. Kloepper J. W. (1997). Bacterial endophytes in agricultural crops can. J. Microbiol. 37, 361–370. doi: 10.1177/0095244305054674

[ref25] Hardoim P. R. Andreote F. D. Reinhold-Hurek B. Sessitsch A. Overbeek L. S. Elsas J. D. (2011). Rice root-associated bacteria: insights into community structures across 10 cultivars. FEMS Microbiol. Ecol. 77, 154–164. doi: 10.1111/j.1574-6941.2011.01092.x, PMID: 21426364 PMC4339037

[ref26] Hardoim P. R. Overbeek L. S. Berg G. Pirttil A. M. Compant S. Campisano A.. (2015). The hidden world within plants: ecological and evolutionary considerations for defining functioning of microbial endophytes. Microbiol. Mol. Biol. Rev. 79, 293–320. doi: 10.1128/MMBR.00050-14, PMID: 26136581 PMC4488371

[ref27] Harman G. Khadka R. Doni F. Uphoff N. (2021). Benefits to plant health and productivity from enhancing plant microbial Symbionts. Front. Plant Sci. 11:610065. doi: 10.3389/fpls.2020.610065, PMID: 33912198 PMC8072474

[ref28] Hassani M. A. Durán P. Hacquard S. (2018). Microbial interactions within the plant holobiont. Microbiome 6, 58. doi: 10.1186/s40168-018-0445-0, PMID: 29587885 PMC5870681

[ref29] Khan M. A. Rahman A. A. Islam S. Khandokhar P. Parvin S. Islam M. B.. (2013). A comparative study on the antioxidant activity of methanolic extracts from different parts of *Morus alba* L. (moraceae). BMC Res. Notes 6, 24. doi: 10.1186/1756-0500-6-24, PMID: 23331970 PMC3559264

[ref30] Kim K. N. Cheong Y. H. Grant J. J. Pandey G. K. Luan S. (2003). *CIPK3*, a calcium sensor-associated protein kinase that regulates abscisic acid and cold signal transduction in Arabidopsis. Plant Cell 15, 411–423. doi: 10.2307/3871874, PMID: 12566581 PMC141210

[ref31] Krivoruchko A. Kuyukina M. Ivshina I. (2019). Advanced *Rhodococcus* biocatalysts for environmental biotechnologies. Catalysts 9, 236. doi: 10.3390/catal9030236

[ref32] Lodewyckx C. Vangronsveld J. Porteous F. Moorea E. R. B. Taghavi S. Mezgeay M.. (2002). Endophytic bacteria and their potential applications. Crit. Rev. Plant Sci. 21, 583–606. doi: 10.1080/0735-260291044377

[ref33] Ma B. Lv X. F. Warren A. Gong J. (2013). Shifts in diversity and community structure of endophytic bacteria and archaea across root, stem and leaf tissues in the common reed, *Phragmites australis*, along a salinity gradient in a marine tidal wetland of northern China. Antonie Van Leeuwenhoek 104, 759–768. doi: 10.1007/s10482-013-9984-3, PMID: 23897211

[ref34] Magoč T. Salzberg S. L. (2011). FLASH: fast length adjustment of short reads to improve genome assemblies. Bioinformatics 27, 2957–2963. doi: 10.1093/bioinformatics/btr507, PMID: 21903629 PMC3198573

[ref35] Mahajan S. Tuteja N. (2005). Cold, salinity and drought stresses: an overview. Arch. Biochem. Biophys. 444, 139–158. doi: 10.1016/j.abb.2005.10.018, PMID: 16309626

[ref36] Mendes L. W. Raaijmakers M. R. Hollander M. Mendes R. Tsai S. M. (2018). Influence of resistance breeding in common bean on rhizosphere microbiome composition and function. ISME J. 12, 212–224. doi: 10.1038/ismej.2017.158, PMID: 29028000 PMC5739014

[ref37] Mishra P. K. Bisht S. C. Ruwari P. Selvakumar G. Joshi G. K. Bisht J. K.. (2011). Alleviation of cold stress in inoculated wheat (*Triticum aestivum L.*) seedlings with psychrotolerant *Pseudomonads* from NW Himalayas. Arch. Microbiol. 193, 497–513. doi: 10.1007/s00203-011-0693-x, PMID: 21442319

[ref38] Moyer C. L. Morita R. Y. (2001). Psychrophiles and Psychrotrophs. Encyclopedia of Life Sciences John Wiley & Sons, Ltd.

[ref39] Nxumalo C. I. Ngidi L. S. Shandu J. S. E. Maliehe T. S. (2020). Isolation of endophytic bacteria from the leaves of *Anredera cordifolia* CIX1 for metabolites and their biological activities. BMC Complement. Med. Therap. 20, 300. doi: 10.1186/s12906-020-03095-z, PMID: 33028279 PMC7541265

[ref40] Ou T. Xu W. F. Wang F. Strobel G. Zhou Z. Y. Xiang Z. H.. (2019). A microbiome study reveals seasonal variation in endophytic bacteria among different mulberry cultivars. Comput. Struct. Biotechnol. J. 17, 1091–1100. doi: 10.1016/j.csbj.2019.07.018, PMID: 31452862 PMC6702411

[ref41] Pan Y. L. Zhang L. (2006). Description Specifications and Data Standards of Mulberry Germplasm. (Beijing: China agriculture press), 62–63.

[ref42] Saeed B. Baranwal V. K. Khurana P. (2016). Comparative transcript omics and comprehensive marker resource development in mulberry. BMC Genomics 17, 16–24. doi: 10.1186/s12864-016-2417-8, PMID: 26846165 PMC4743097

[ref43] Saranraj P. Sivasakthivelan P. Al-Tawaha A. R. M. Sudha A. Al-Tawaha A. R. Sirajuddin S. N.. (2021). Diversity and evolution of Bradyrhizobium communities relating to soybean cultivation: a review. IOP Conf. Ser. Earth Environ. Sci. 788:012208. doi: 10.1088/1755-1315/788/1/012208

[ref44] Selvakumar G. Joshi P. Suyal P. Mishra P. K. Joshi G. K. Bisht J. K.. (2011). *Pseudomonas lurida* M2RH3 (MTCC 9245), a psychrotolerant bacterium from the Uttarakhand Himalayas, solubilizes phosphate and promotes wheat seedling growth. World J. Microbiol. Biotechnol. 27, 1129–1135. doi: 10.1007/s11274-010-0559-4

[ref45] Shi Y. W. Yang H. M. Zhang T. Sun J. Lou K. (2014). Illumina-based analysis of endophytic bacterial diversity and space-time dynamics in sugar beet on the north slope of Tianshan mountain. Appl. Microbiol. Biotechnol. 98, 6375–6385. doi: 10.1007/s00253-014-5720-9, PMID: 24752839

[ref46] Shukla P. Rohela G. K. Shabnam A. A. Sharma S. P. (2016). Prospect of cold tolerant genes and its utilization in mulberry improvement. Indian Hortic. J. 6, 127–129.

[ref47] Singh L. P. Gill S. S. Tuteja N. (2011). Unraveling the role of fungal symbionts in plant abiotic stress tolerance. Plant Signal. Behav. 6, 175–191. doi: 10.4161/psb.6.2.14146, PMID: 21512319 PMC3121976

[ref48] Solanke A. U. Sharma A. K. (2008). Signal transduction during cold stress in plants. Physiol. Mol. Biol. Plants 14, 69–79. doi: 10.1007/s12298-008-0006-2, PMID: 23572874 PMC3550661

[ref49] Stackebrandt E. Goebel B. M. (1994). Taxonomic note: a place for DNA-DNA reassociation and 16S rRNA sequence analysis in the present species definition in bacteriology. Int. J. Syst. Bacteriol. 44, 846–849. doi: 10.1099/00207713-44-4-846

[ref50] Steponkus P. L. (1984). Role of the plasma membrane in freezing injury and low temperature acclimation. Annu. Rev. Plant Physiol. 35, 543–584. doi: 10.1146/annurev.pp.35.060184.002551

[ref51] Steponkus P. L. Uemura M. Webb M. S. (1993). “A contrast of the cryostability of the plasma membrane of winter rye and spring oat-two species that widely differ in their freezing tolerance and plasma membrane lipid composition,” in Advances in Low-Temperature Biology. *Vol. 2.* ed. Steponkus P. L. (London: JAI Press), 211–312.

[ref52] Theocharis A. Bordiec S. Fernandez O. Paquis S. Dhondt-Cordelier S. Baillieul F.. (2012). *Burkholderia phytofirmans* PsJN primes *Vitis vinifera*L. and confers a better tolerance to low nonfreezing temperatures. Mol. Plant Microbe Interact. J. 25, 241–249. doi: 10.1094/MPMI-05-11-0124, PMID: 21942451

[ref53] Tian X. Y. Zhang C. S. (2017). Illumina-based analysis of endophytic and rhizosphere bacterial diversity of the coastal halophyte *Messerschmidia sibirica*. Front. Microbiol. 8:2288. doi: 10.3389/fmicb.2017.02288, PMID: 29209296 PMC5701997

[ref54] Ukaji N. Kuwabara C. Takezawa D. Arakawa K. Fujikawa S. (2004). Accumulation of pathogenesis-related (PR) 10/bet v 1 protein homologues in mulberry (*Moru*s bombycis Koidz.) tree during winter. Plant Cell Environ. 27, 1112–1121. doi: 10.1111/j.1365-3040.2004.01216.x

[ref55] Vandenkoornhuyse P. Quaiser A. Duhamel M. Le Van A. Dufresne A. (2015). The importance of the microbiome of the plant holobiont. New Phytol. 206, 1196–1206. doi: 10.1111/nph.13312 25655016

[ref56] Wang Q. Garrity G. M. Tiedje J. M. Cole J. R. (2007). Naive Bayesian classifier for rapid assignment of rRNA sequences into the new bacterial taxonomy. Appl. Environ. Microbiol. 73, 5261–5267. doi: 10.1128/AEM.00062-07, PMID: 17586664 PMC1950982

[ref57] Wei X. T. Jiang F. G. Han B. Zhang H. Huang D. Shao X. Q.. (2021). New insight into the divergent responses of plants to warming in the context of root endophytic bacterial and fungal communities. Peer J. 9:e11340. doi: 10.7717/peerj.11340, PMID: 34123582 PMC8164412

[ref58] Xie J. Xia T. (2008). Research of mulberry endophytes and the prospect of their application. Newslett. Sericult. Sci. 28, 44–47.

[ref59] Xin Y. H. Zhou Y. G. Dong X. Z. (2013). Biodiversity and cold adaptive mechanisms of psychrophiles. Biodivers. Sci. 21, 468–480. doi: 10.3724/SP.J.1003.2013.13040

[ref60] Yadav A. N. Sachan S. G. Verma P. Saxena A. K. (2014). Prospecting cold deserts of North-Western Himalayas for microbial diversity and plant growth promoting attributes. J. Biosci. Bioeng. 119, 683–693. doi: 10.1016/j.jbiosc.2014.11.006 25575970

[ref61] Yadav A. N. Sachan S. G. Verma P. Saxena A. K. (2016). Bioprospecting of plant growth promoting psychrotrophic Bacilli from the cold desert of north-western Indian Himalayas. Indian J. Exp. Biol. 54, 142–150. PMID: 26934782

[ref62] Yadav A. N. Sachan S. G. Verma P. Tyagi S. P. Kaushik R. Saxena A. K. (2015). Culturable diversity and functional annotation of psychrotrophic bacteria from cold desert of Leh Ladakh (India). World J. Microbiol. Biotechnol. 31, 95–108. doi: 10.1007/s11274-014-1768-z, PMID: 25371316

[ref63] Yang R. X. Liu P. Ye W. Y. (2017). Illumina-based analysis of endophytic bacterial diversity of tree peony (Paeonia sect. Moutan) roots and leaves. Braz. J. Microbiol. 48, 695–705. doi: 10.1016/j.bjm.2017.02.009, PMID: 28606427 PMC5628320

[ref64] Zhou L. Li C. White J. F. Johnson R. D. (2021). Synergism between calcium nitrate applications and fungal endophytes to increase sugar concentration in *Festuca sinensis* under cold stress. Peer J. 9:e10568. doi: 10.7717/peerj.10568, PMID: 35070512 PMC8759379

