# Supplementary material for: Diversity of endophytic bacteria of mulberry (Morus L.) under cold conditions
Source: Front Microbiol. 2022 Jul 19;13:923162. doi: 10.3389/fmicb.2022.923162 (PMC9344060; doi:10.3389/fmicb.2022.923162)
Supplement: Supplementary file 1 [file Data_Sheet_1.docx]

Supplementary Material

**Table S2 Characteristics of effective tags from samples of endophytic bacteria associated with Mulberry**

| Sample | Repeat | Seq_num | Base_num | Mean_length |
| --- | --- | --- | --- | --- |
| JSDS | 1 | 41924 | 15747375 | 376 |
|  | 2 | 46572 | 17493071 | 376 |
|  | 3 | 38168 | 14325466 | 375 |
| JS792 | 1 | 33405 | 12532489 | 375 |
|  | 2 | 34571 | 12985029 | 376 |
|  | 3 | 32076 | 12059437 | 376 |
| JRDS | 1 | 33040 | 12444186 | 377 |
|  | 2 | 44936 | 16937777 | 377 |
|  | 3 | 40198 | 15145641 | 377 |
| JR792 | 1 | 40088 | 15126654 | 377 |
|  | 2 | 38900 | 14666310 | 377 |
|  | 3 | 46413 | 17504902 | 377 |
| FSDS | 1 | 37296 | 14074409 | 377 |
|  | 2 | 53938 | 20352416 | 377 |
|  | 3 | 41192 | 15525969 | 377 |
| FS792 | 1 | 30762 | 11603757 | 377 |
|  | 2 | 44669 | 16848426 | 377 |
|  | 3 | 42873 | 16168583 | 377 |
| FRDS | 1 | 60754 | 22865489 | 376 |
|  | 2 | 71613 | 27002760 | 377 |
|  | 3 | 57108 | 21511411 | 377 |
| FR792 | 1 | 48566 | 18291289 | 377 |
|  | 2 | 53586 | 20209443 | 377 |
|  | 3 | 47095 | 17752550 | 377 |

**Table S3 Percentage of dominance phyla for different samples**

|  | Proteobacteria | Actinobacteria | Firmicutes |
| --- | --- | --- | --- |
| JSDS | 43.64±4.36Cd | 45.88±3.69Aa | 7.08±1.03Aa |
| JS792 | 47.65±1.92Cd | 43.45±2.03Aa | 6.36±0.71Aa |
| JRDS | 53.32±4.89BCcd | 44.07±4.86Aa | 0.06±0.07Bb |
| JR792 | 59.38±2.47BCbc | 38.02±2.35ABab | 0.16±0.12Bb |
| FSDS | 88.44±1.78Aa | 9.6±1.31Cc | 0.79±0.39Bb |
| FS792 | 86.18±1.88Aa | 10.93±1.92Cc | 0.55±0.13Bb |
| FRDS | 59.56±3.93BCbc | 36.97±2.94ABab | 0.08±0.03Bb |
| FR792 | 68.99±3.35Bb | 28.16±4.19Bb | 0.1±0.09Bb |

**Table S4 Percentage of dominance genera for different samples**

|  | JSDS | JS792 | JRDS | JR792 | FSDS | FS792 | FRDS | FR792 |
| --- | --- | --- | --- | --- | --- | --- | --- | --- |
| *Pseudomonas* | 3.36±1.23Bbc | 4.21±2.61Bbc | 0.03±0.06Bc | 0.08±0.04Bc | 73.14±17.74Aa | 70.31±6.69Aa | 2.09±1.45Bbc | 11.02±4.41Bb |
| *Steroidobacter* | 0.4±0.98BCb | 0.36±0.25BCb | 36.08±27.46Aa | 29.96±13.34ABa | 0.21±0.5Cb | 0.64±1.45BCb | 43.37±27.8Aa | 27.62±17.61ABCa |
| *Rhodococcus* | 26.16±13.51Aa | 34.63±9.4Aa | 0.16±0.08Bb | 0.27±0.37Bb | 7.09±6.84Bb | 6.61±1.93Bb | 0.21±0.19Bb | 0.83±0.29Bb |
| *Ralstonia* | 23.2±9.6Aa | 26.37±10.25Aa | 0.14±0.05Bb | 0.22±0.29Bb | 6±4.73Bb | 5.68±0.57Bb | 0.2±0.05Bb | 0.9±0.63Bb |
| *Cryptosporangium* | 0.02±0.04Bb | 0.08±0.04Bb | 16.25±18.84Aa | 5.89±10.26ABab | 0.02±0.05Bb | 0.18±0.36Bb | 11.61±13.28ABab | 1.76±4.44ABb |
| *Bradyrhizobium* | 0.1±0.15Bc | 0.24±0.29Bc | 4.91±4.8ABabc | 11.68±9.97Aa | 0.02±0.01Bc | 0.21±0.27Bc | 4.37±5.35ABbc | 8.38±3.13ABab |
| *Acidibacter* | 0.02±0.05Ab | 0.18±0.38Ab | 0.86±1Aab | 3.92±6.92Aab | 0.01±0.02Ab | 0.11±0.12Ab | 0.33±0.21Ab | 4.38±3.06Aa |
| *Bacillus* | 3.69±2.82Aa | 3.73±3.61Aa | 0.03±0.02Bb | 0.09±0.17Bb | 0.02±0.04Bb | 0.13±0.14Bb | 0.03±0.03Bb | 0.04±0.05Bb |
| *Sphingomonas* | 3.97±4.33Aa | 1.64±3.73Aab | 0.36±0.3Ab | 0.43±0.37Ab | 0.45±1.07Ab | 0.26±0.32Ab | 0.18±0.16Ab | 0.11±0.25Ab |
| *Frigoribacterium* | 6.05±6.64Aa | 0.24±0.58Bb | 0±0Bb | 0±0Bb | 0.18±0.21Bb | 0±0Bb | 0±0Bb | 0±0Bb |
| *Pseudokineococcus* | 3.35±3.29Aa | 0.52±0.93Bb | 0±0Bb | 0±0Bb | 0.16±0.19Bb | 0±0Bb | 0±0Bb | 0±0Bb |

**Table S5 Top 5 dominant genera of different samples**

|  | JSDS | JS792 | JRDS | JR792 | FSDS | FS792 | FRDS | FR792 |
| --- | --- | --- | --- | --- | --- | --- | --- | --- |
| *1* | *Rhodococcus* | *Rhodococcus* | *Steroidobacter* | *Steroidobacter* | *Pseudomonas* | *Pseudomonas* | *Steroidobacter* | *Steroidobacter* |
| *2* | *Ralstonia* | *Ralstonia* | *Cryptosporangium* | *Bradyrhizobium* | *Rhodococcus* | *Rhodococcus* | *Cryptosporangium* | *Pseudomonas* |
| *3* | *Frigoribacterium* | *Pseudomonas* | *Mycobacterium* | *Actinomadura* | *Ralstonia* | *Ralstonia* | *Mycobacterium* | *Bradyrhizobium* |
| *4* | *Sphingomonas* | *Methylobacterium* | *Virgisporangium* | *Mycobacterium* | *unclassified_p__Proteobacteria* | *unclassified_p__Proteobacteria* | *Bradyrhizobium* | *Mycobacterium* |
| *5* | *Bacillus* | *Bacillus* | *Bradyrhizobium* | *Cryptosporangium* | *g__Sphingomonas* | *Steroidobacter* | *Virgisporangium* | *Virgisporangium* |

**Table S6 Significance of different pathways**

| Sample1 | Sample2 | Metabolism | Environmental Information Processing | Cellular Processes | Genetic Information Processing | Human Diseases | Organismal Systems |
| --- | --- | --- | --- | --- | --- | --- | --- |
| JSDS | JS792 | _ | * | _ | _ | _ | _ |
| JRDS | JR792 | _ | _ | _ | _ | _ | _ |
| FSDS | FS792 | _ | _ | _ | _ | _ | _ |
| FRDS | FR792 | _ | _ | _ | _ | _ | _ |
| JSDS | FSDS | * | *** | *** | ** | *** | ** |
| JS792 | FS792 | *** | *** | *** | *** | *** | *** |
| JRDS | FRDS | * | *** | *** | _ | *** | _ |
| JR792 | FR792 | _ | _ | _ | _ | _ | _ |
| JSDS | JRDS | _ | _ | _ | _ | _ | _ |
| JS792 | JR792 | _ | _ | _ | ** | _ | _ |
| FSDS | FRDS | * | *** | *** | * | *** | * |
| FS792 | FR792 | *** | *** | *** | *** | ** | *** |

**
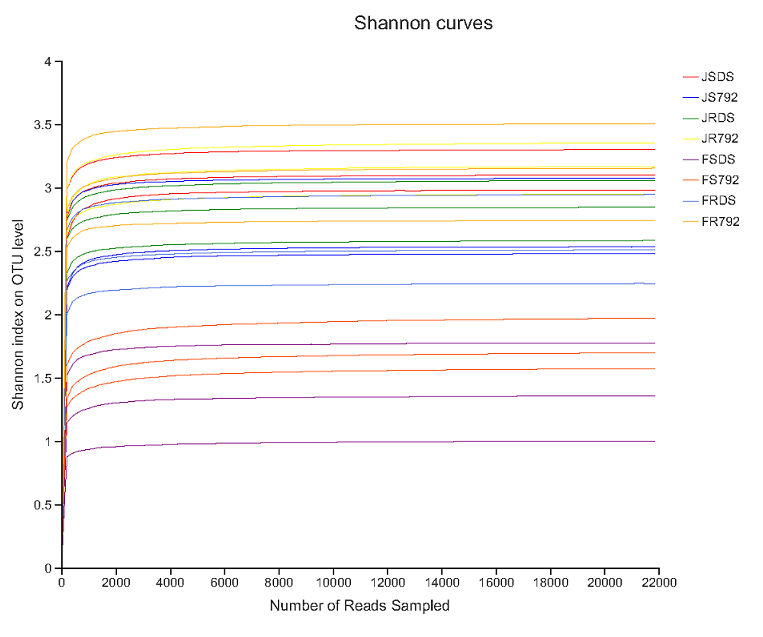
**

**Fig. S1 Rarefaction curves depicting the number of OTUs with 97% similarity identified from different samples.**

The x-axis represents the number of sequences obtained from each library and the y-axis represents the number of observed OTUs. JSDS, JRDS, JS792 and JR792 represent bacterial communities from the stem and root of ‘Da shi’ and ‘Xuan No.792’ in January, respectively. FSDS, FRDS, FS792 and FR792 represent bacterial communities from the stem and root of ‘Da shi’ and ‘Xuan No.792’ in February, respectively.


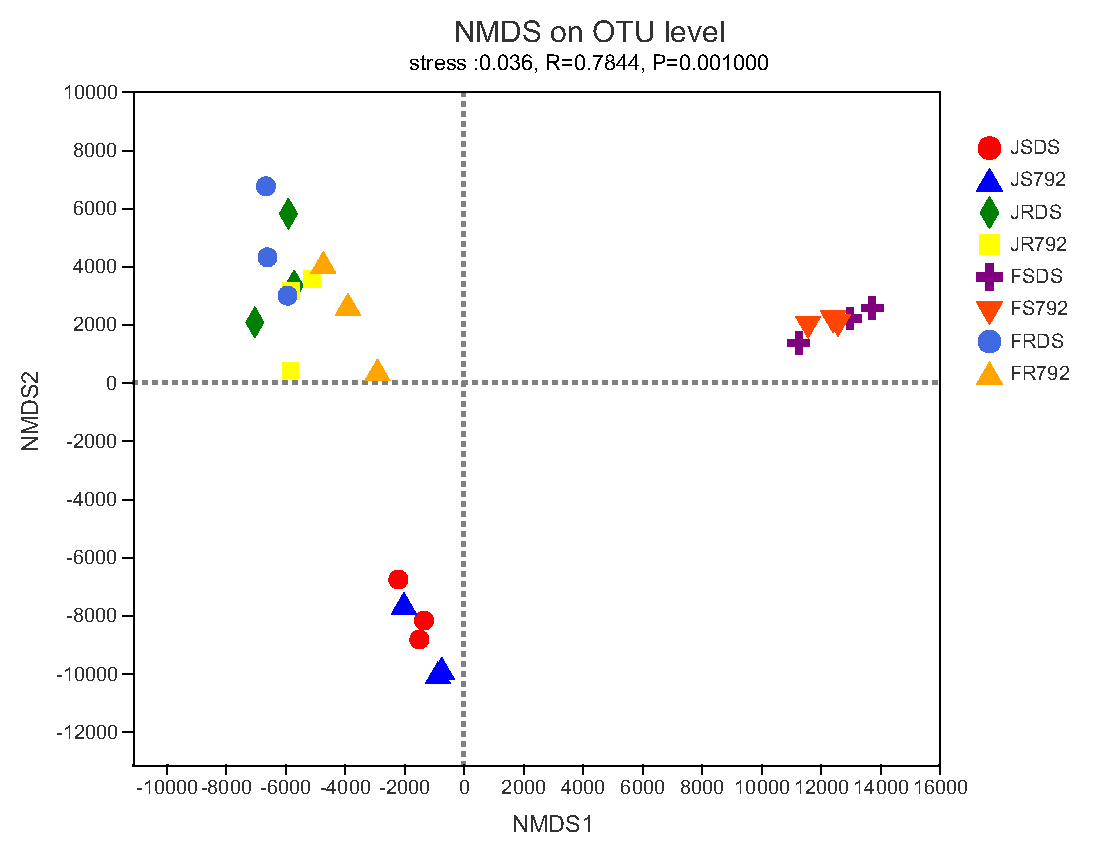


**FigS2 NMDS of the relationship between samples on the basis of similarity in the community composition of bacterial OTUs.**

**Table S7 The unique and shared OTUs of root and stem**

|  |  | Root(%) | Share(%) | Stem (%) |
| --- | --- | --- | --- | --- |
| DS | January | 42.53 | 19.37 | 38.11 |
|  | February | 35.84 | 25.96 | 38.20 |
| 792 | January | 50.89 | 22.93 | 26.18 |
|  | February | 14.58 | 31.17 | 54.25 |

**Table S8 The unique and shared OTUs of DS and X792**

|  |  | DS(%) | Share(%) | X792(%) |
| --- | --- | --- | --- | --- |
| Stem | January | 35.11 | 35.89 | 29.00 |
|  | February | 13.90 | 27.23 | 58.86 |
| Root | January | 18.66 | 45.12 | 36.23 |
|  | February | 23.08 | 43.03 | 33.89 |

**Table S9 The unique and shared OTUs of January and February**

|  |  | January(%) | Share(%) | February(%) |
| --- | --- | --- | --- | --- |
| Stem | DS | 30.28 | 36.39 | 33.33 |
|  | 792 | 13.09 | 23.20 | 63.71 |
| Root | DS | 29.76 | 46.62 | 23.63 |
|  | 792 | 32.20 | 47.25 | 20.55 |

**Table S10 Pathway level1**

|  | JSDS | JS792 | p-value |
| --- | --- | --- | --- |
| Metabolism | 60017655 | 65005339 | 0.0632 |
| Environmental Information Processing | 4315497 | 4657908 | 0.0100 |
| Cellular Processes | 3223259 | 3393486 | 0.1437 |
| Genetic Information Processing | 3888536 | 3870584 | 0.8975 |
| Human Diseases | 2447155 | 2606490 | 0.1580 |
| Organismal Systems | 1556549 | 1715448 | 0.0567 |

**Table S11 Pathway level2**

|  |  | X792 | DS | p-value |
| --- | --- | --- | --- | --- |
| Metabolism | Energy metabolism | 3418892 | 3154754 | 0.041898 |
| Environmental Information Processing | Membrane transport | 2578211 | 2397416 | 0.01286 |
| Environmental Information Processing | Signal transduction | 2079613 | 1918041 | 0.013209 |
| Cellular Processes | Cellular community - prokaryotes | 1896561 | 1772208 | 0.018184 |
| Human Diseases | Infectious disease: bacterial | 463846.8 | 434817.3 | 0.005005 |
| Human Diseases | Endocrine and metabolic disease | 170484.4 | 160273.1 | 0.036244 |
| Human Diseases | Substance dependence | 41300.46 | 32589.65 | 0.014834 |

Complete data sets were submitted to the NCBI Short Read Archive (SRA) database (Accession Number:

SRR18790631, SRR18790632, SRR18790633, SRR18790634, SRR18790635, SRR18790636, SRR18790637, SRR18790638, SRR18790639, SRR18790640, SRR18790641, SRR18790642, SRR18790643, SRR18790644, SRR18790645, SRR18790646, SRR18790647, SRR18790648, SRR18790649, SRR18790650, SRR18790651, SRR18790652, SRR18790653, SRR18790654
